# Functional Monitoring of Patients With Knee Osteoarthritis Based on Multidimensional Wearable Plantar Pressure Features: Cross-Sectional Study

**DOI:** 10.2196/58261

**Published:** 2024-11-25

**Authors:** Junan Xie, Shilin Li, Zhen Song, Lin Shu, Qing Zeng, Guozhi Huang, Yihuan Lin

**Affiliations:** 1 School of Microelectronics South China University of Technology Guangzhou China; 2 The First Affiliated Hospital of Nanchang University Nanchang China; 3 Department of Biomedical Engineering Hong Kong Polytechnic University Hong Kong China (Hong Kong); 4 School of Future Technology South China University of Technology Guangzhou China; 5 Zhongshan Institute of Modern Industrial Technology of South China University of Technology Zhongshan China; 6 Department of Rehabilitation Medicine Zhujiang Hospital Southern Medical University Guangzhou China; 7 School of Rehabilitation Southern Medical University Guangzhou China; 8 School of Electronic and Information Engineering South China University of Technology Guangzhou China

**Keywords:** knee osteoarthritis, KOA, 40-m fast-paced walk test, 40mFPWT, timed up-and-go test, TUGT, timed up and go, TUG, functional assessment, monitoring, wearable, gait, walk test, plantar, knee, joint, arthritis, gait analysis, regression model, machine learning

## Abstract

**Background:**

Patients with knee osteoarthritis (KOA) often present lower extremity motor dysfunction. However, traditional radiography is a static assessment and cannot achieve long-term dynamic functional monitoring. Plantar pressure signals have demonstrated potential applications in the diagnosis and rehabilitation monitoring of KOA.

**Objective:**

Through wearable gait analysis technology, we aim to obtain abundant gait information based on machine learning techniques to develop a simple, rapid, effective, and patient-friendly functional assessment model for the KOA rehabilitation process to provide long-term remote monitoring, which is conducive to reducing the burden of social health care system.

**Methods:**

This cross-sectional study enrolled patients diagnosed with KOA who were able to walk independently for 2 minutes. Participants were given clinically recommended functional tests, including the 40-m fast-paced walk test (40mFPWT) and timed up-and-go test (TUGT). We used a smart shoe system to gather gait pressure data from patients with KOA. The multidimensional gait features extracted from the data and physical characteristics were used to establish the KOA functional feature database for the plantar pressure measurement system. 40mFPWT and TUGT regression prediction models were trained using a series of mature machine learning algorithms. Furthermore, model stacking and average ensemble learning methods were adopted to further improve the generalization performance of the model. Mean absolute error (MAE), mean absolute percentage error (MAPE), and root mean squared error (RMSE) were used as regression performance metrics to evaluate the results of different models.

**Results:**

A total of 92 patients with KOA were included, exhibiting varying degrees of severity as evaluated by the Kellgren and Lawrence classification. A total of 380 gait features and 4 physical characteristics were extracted to form the feature database. Effective stepwise feature selection determined optimal feature subsets of 11 variables for the 40mFPWT and 10 variables for the TUGT. Among all models, the weighted average ensemble model using 4 tree-based models had the best generalization performance in the test set, with an MAE of 2.686 seconds, MAPE of 9.602%, and RMSE of 3.316 seconds for the prediction of the 40mFPWT and an MAE of 1.280 seconds, MAPE of 12.389%, and RMSE of 1.905 seconds for the prediction of the TUGT.

**Conclusions:**

This wearable plantar pressure feature technique can objectively quantify indicators that reflect functional status and is promising as a new tool for long-term remote functional monitoring of patients with KOA. Future work is needed to further explore and investigate the relationship between gait characteristics and functional status with more functional tests and in larger sample cohorts.

## Introduction

Knee osteoarthritis (KOA) is a degenerative and irreversible joint disease with typical symptoms including pain, stiffness, decreased joint mobility, and gait disturbance [1,2]. These symptoms worsen with the progression of the disease and may lead to serious treatment consequences, such as total knee replacement and the need for corresponding gait correction rehabilitation training. In recent years, the number of individuals diagnosed with KOA has rapidly increased owing to the aging of the global population and the rising prevalence of obesity. KOA is the primary cause of dysfunction among older adults, placing an extensive burden on both socioeconomic and medical systems [3]. Assessment of physical function in KOA is a crucial component of documenting and evaluating rehabilitation progress [4,5], which will accelerate the establishment of new diagnostic criteria and effective rehabilitation methods [6-8]. Traditional radiography presents limitations as a static assessment tool and cannot achieve long-term dynamic functional monitoring [9]. Both patient-reported outcomes (PROs) and performance-based measures (PBMs) have been used to assess physical function in KOA, but there is no universally recognized gold standard for evaluation [10]. Although PROs are convenient and cost-effective, their high subjectivity and susceptibility to patient’s pain and emotions can lead to biased results. Moreover, they are not suitable for individuals with depression or cognitive impairment [5,11]. PBMs are objective and effective evaluation methods, considering factors such as time, cost, equipment, space, and management burden comprehensively. The OARSI (Osteoarthritis Research Society International) recommended a set of performance-based physical function tests, such as the 40-m fast-paced walk test (40mFPWT) and timed up-and-go test (TUGT) [12], whose validity and reliability have been validated by many research reports [13]. However, PBMs still need to be conducted in specific locations, such as hospitals or rehabilitation clinics, and under the supervision of well-trained medical practitioners. Therefore, developing a simple, fast, effective, and user-friendly method for functional assessment is beneficial to relieve the burden on the health care system in society.

Researchers are now able to easily acquire vast amounts of biomedical data through wearable sensors, including pressure sensors [14], inertial measurement units [15,16], and electromyography sensors [17]. The application of machine learning technology for the analyzing and processing of this data facilitates the identification and prediction of human physiological conditions and disease risks [18], thereby offering new opportunities for attaining personalized health care and health management [19]. Plantar pressure signal has shown potential applications in the diagnosis and rehabilitation evaluation of KOA. Studies [20-24] have shown that patients with KOA are prone to abnormal gait or gait dysfunction due to pain, stiffness, limited joint range of motion, and other symptoms, and their gait patterns are specifically characterized by unstable gait and high variability. Naili et al [1] found gait deviations between patients with KOA and healthy population through 3D gait analysis and suggested that PBMs may be more closely associated with overall gait pattern deviations in patients with KOA than PROs or perceived pain. In addition, statistical analysis of foot pressure parameters measured by the F-Scan (Tekscan, Inc) system showed that the pressure in the thumb and heel area of patients with KOA as a percentage of weight was significantly lower than that in healthy people, but the central region was higher [25,26]. Moreover, the center of pressure (COP) path range was smaller in the KOA group than in the healthy group, which may be due to incomplete gait in patients with KOA [27].

Unlike inertial measurement units, plantar pressure sensors provide stable and accurate plantar pressure distribution data, which is essential for accurate gait analysis, without being affected by changes in the wearing position or method. In contrast to force platforms, instrumented treadmills, and 3D gait analysis technologies [28], footwear systems with embedded foot pressure sensors can overcome the limitations of laboratory settings and enable long-term remote monitoring by inconspicuously integrating them into everyday footwear. Previous work on the functional evaluation of KOA using wearable pressure sensors has focused on PROs such as the Western Ontario and McMaster Universities Osteoarthritis Index (WOMAC) [19,29] and Knee Osteoarthritis Outcome Score [8]. However, the connection between KOA gait characteristics and more objective PBMs has rarely been explored. Table 1 summarizes the differences between important relevant studies and ours per methods, gait features, and objectives.

Therefore, in this study, a wireless in-shoe system integrating a low-cost, high-durability foot pressure sensor was used to collect plantar pressure data during walking for patients with KOA. The performance of the shoe system used has been validated in previous research, demonstrating its ability to effectively monitor human gait dynamics information in real time for daily use, and has been applied to the detection of diabetic feet [30] and fall risk assessment studies in older adults [31,32]. We suggest that there is a mapping correlation between functional performance and gait features in patients with KOA. From the raw plantar pressure data, spatiotemporal parameters were extracted to construct a KOA gait feature database customized for wearable plantar pressure sensors. This construction involved expanding the dimensionality of gait features through mathematical methodologies. Effective feature selection and analysis were performed for the 40mFPWT and TUGT tasks, respectively. The objective of this study is to develop a functional evaluation model using multidimensional plantar pressure features to monitor and assess the functional performance of patients with KOA, potentially serving as a self-managed rehabilitation tool to provide long-term remote dynamic functional monitoring and progress recording for patients with KOA [33].

**Table 1:** Review of related works.

References	Methods	Gait features	Objectives
Kwon et al [19]	3D gait analysis and machine learning	Kinetic, kinematic, and spatial-temporal data	Develop estimation models for WOMAC^a^ scores of patients with KOA^b^
Ofran et al [28]	3D gait analysis and multiple regression analysis	Spatiotemporal gait parameters	Predict common functional tests by spatiotemporal gait parameters in patients post stroke
Wada et al [24]	IMUs^c^ and statistical analysis	Scalar product and time features	Clarify the gait characteristics of patients with KOA
Saito et al [25]	Pressure sensors (F-Scan) and statistical analysis	Walking speed, COP^d^, %PFP^e^, %Long^f^, %Trans^g^, navicular height ratio, etc.	Clariﬁed foot pressure patterns and hindfoot deformities in KOA and analyzed their associations with foot pain
Ours	Pressure sensors and machine learning	Multidimensional wearable plantar pressure features	Develop a functional assessment model for PBMs^h^ scores of patients with KOA

^a^WOMAC: Western Ontario and McMaster Universities Osteoarthritis Index.

^b^KOA: knee osteoarthritis.

^c^IMU: inertial measurement units.

^d^COP: center of pressure.

^e^%PFP: partial foot pressure as the percentage of body weight.

^f^%Long: anteroposterior length of the center of pressure path as a percentage of foot length.

^g^%Trans: transverse width of the center of pressure path as the percentage of foot width.

^h^PBM: performance-based measure.

## Methods

### Recruitment and Data Collection

The research enlisted 92 adults diagnosed clinically as patients with KOA, exhibiting varying degrees of severity as evaluated by the Kellgren and Lawrence classification. These participants demonstrated independent walking capability for a duration of 2 minutes. All participants were sourced from Zhujiang Hospital of Southern Medical University, and the tests were administered under the guidance of proficiently trained medical personnel.

Participants were thoroughly briefed on the procedures and paradigm of the functional tests before they underwent the assessments. Adequate intervals were implemented between each test session to avoid the impact of fatigue. Successively, the participants underwent the TUGT and 40mFPWT to respectively evaluate the patients’ overall functional mobility, balance capacity, short-distance walking performance, and gait speed. The TUGT and 40mFPWT scores indicate the time taken to complete the test, with higher scores representing worse patient function. Table 2 summarizes the participants’ demographic characteristics and physical function tests. The results of the Mann-Whitney *U* tests revealed no statistical difference between male and female groups in the 40mFPWT and TUGT outcomes (*P*=.48 and *P*=.50, respectively).

**Figure 1:**
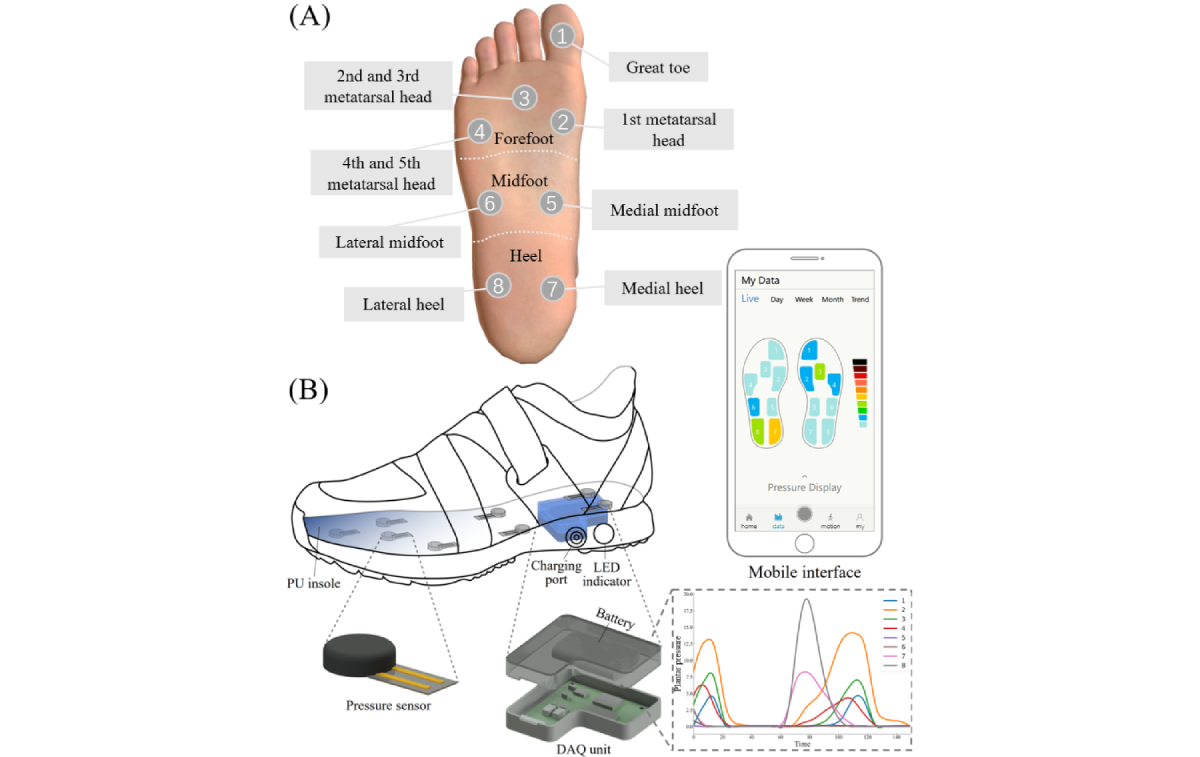
(A) Location of sensor deployment. (B) The composition of plantar pressure shoe measurement system. DAQ: data acquisition; PU: polyurethane.

The footwear system used for the collection of plantar pressure data has been detailed in prior studies [30]. Each shoe is equipped with eight integrated pressure sensors capable of detecting vertical ground reaction forces during walking. The sensor position distributions and corresponding pressure-sensing regions are illustrated in Figure 1A. Before formal data collection, participants were required to wear suitable socks and shoes. Ample time was provided for participants to adjust and ensure proper fitting of the shoes before engaging in natural walking. The participants were then asked to walk independently back and forth in a 20 m corridor at a freely walking speed for 2 minutes to simulate everyday locomotor activities. The shoe system collected plantar pressure signal data during walking at a frequency of 20 Hz and transmitted it to a mobile phone in real time via Bluetooth (Bluetooth Special Interest Group; Figure 1B).

**Table 2:** Participants’ characteristics.

Variable		Values
**Age (years), mean (SD)**		62.95 (8.4)
**Gender, n**		
	Male	17
	Female	75
**Height (cm), mean (SD)**		158.08 (7.85)
**Weight (kg), mean (SD)**		61.33 (10.47)
**BMI (kg/m²), mean (SD)**		24.51 (3.43)
**TUGT^a^(s), mean (SD)**		11.39 (2.99)
**40mFPWT^b^** **(s), mean (SD)**		28.61 (6.19)

^a^TUGT: timed up-and-go test.

^b^40mFPWT: 40-m fast-paced walk test.

### Feature Extraction

#### Overview

**Figure 2:**
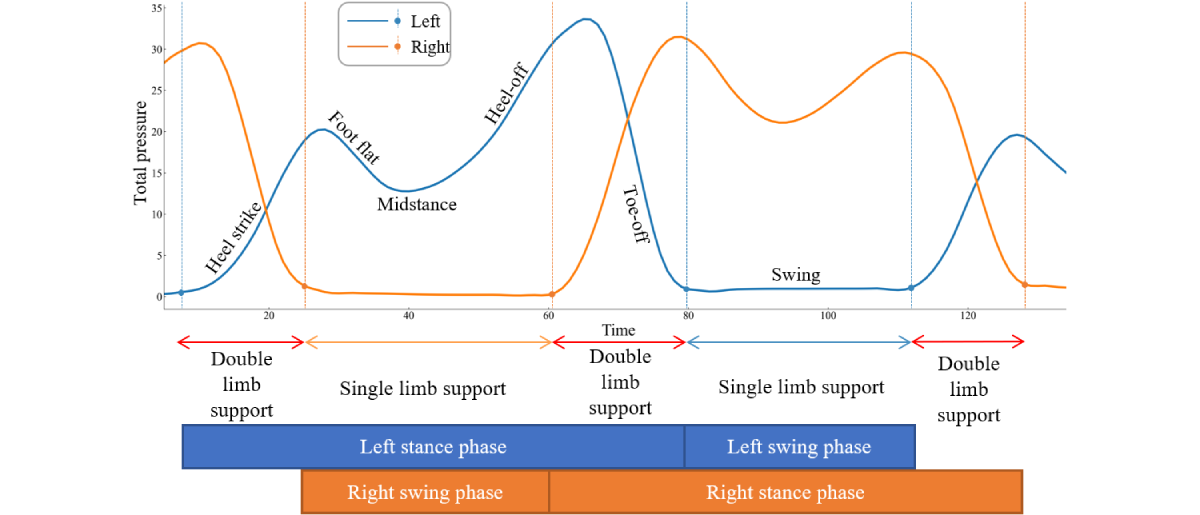
The total VGRF of the left and right feet in a gait cycle and the segmentation points of gait stages are shown. VGRF: vertical ground reaction forces.

Feature extraction is a crucial step in gait data analysis and should adhere to the following principles. First, the features should have clear biomechanical meaning and be objectively observable. This ensures the physiological relevance of the extracted variables. Second, these features should exhibit generality across all participant types. The second principle is particularly pivotal because generalization directly affects the robustness of machine learning techniques [34]. Human gait is a periodic activity composed of the stance phase and the swing phase. In the stance phase, the pressure sensors in the corresponding regions of the heel, middle foot, and forefoot are activated successively as the gait advances. Before feature extraction, gait cycle segmentation is performed on the acquired temporal signal data of plantar pressure. A single gait cycle is divided by identifying the rise point of the total pressure curve from the 8 sensors when the heel strikes the ground, representing a gait cycle from heel strike to the end of the swing phase. When the toe is off the ground, the total pressure curve drops to its minimum value as the dividing point of the terminal stance and preswing phases, as shown in Figure 2. Excluding the starting step of the first 2 steps, the next 50 gait cycles with the left and right feet adjacent were selected as a sample for feature extraction and analysis.

Multidimensional features would be extracted based on the weight-normalized plantar pressure data obtained by the shoe system. The basic features include the single-foot feature extracted for each foot and the bipedal feature. Then the symmetry coefficient feature, SD feature, and the weak foot feature were calculated based on the single foot feature, which greatly enrich the plantar pressure feature database. We refer to some common plantar pressure feature extraction methods reported in previous studies [25,30,31] and perform corresponding feature extraction according to the sensor deployment position of our plantar pressure shoe measurement system. In addition, we not only focus on the extreme value features of a single sensing unit but also analyze the plantar pressure features corresponding to the transition of different stages in the gait cycle. The relevant features of the single sensing region and the combined sensing region corresponding to the subphases of the stance phase were extracted.

#### Peak Plantar Pressure

Peak plantar pressure (PPP) represents the maximum load on the underfoot area during one step. Taking the left foot as an example, the calculation formula for each sensor’s PPP is expressed as equation 1. The maximum pressure values for the eight regions of both the left and right feet are extracted respectively within one step. Here, s ∈ (1,8) denotes the sensor number, P_s_(n) represents the pressure data sequence of the corresponding sensor s, and n indicates the time sampling points. L_1-4_PPP, L_5-6_PPP, and L_7-8_PPP represent the peak pressure combination of the sensors corresponding to the left forefoot, middle foot, and heel in one gait cycle, respectively. The total vertical ground reaction forces in a gait cycle exhibit a bimodal pattern, where the first vertical force peak (L_peak1) is caused by a heel strike, and the second peak (L_peak2) is attributed to the forward movement of the center of gravity during walking. The L_4/2_PPP and L_8/7_PPP represents the ratio of peak pressure in the lateral and medial regions of the left forefoot and heel, respectively. In this part, a total of 30 left and right foot features were extracted.



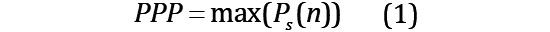



#### Pressure Gradient

Pressure gradient (PG) quantifies the rate of change of pressure over time, reflecting the rapidity of pressure curve fluctuations beneath the foot during locomotion. Positive PG indicates rapid pressure rises during foot-floor contact phases, while negative PG corresponds to swift pressure declines. The maximum and minimum PG of each sensing curve of both feet can be calculated by equations 2 and 3, where Δt is the sampling time interval. Partial region combination sensing can be used to describe the characteristics of state transition between substages of the gait cycle. For instance, L_1-4_MaxPG represents the maximum gradient change upward when the center of gravity shifts to the forefoot of the left foot. On the other hand, L_1-4_MinPG signifies the maximum negative gradient change when the toes lift off the ground, corresponding to the downward pressure curve. The L_5-6_MaxPG and L_5-6_MinPG in the midfoot region reflect the pressure change during the foot flat and heel-off phases, respectively. Similarly, L_7-8_MaxPG and L_7-8_MinPG in the heel region denote the changes during the heel strike and foot flat phases, respectively. The loading rate (L_loadr) is PG calculated by the first peak pressure and the initial contact pressure of the stance phase. The off-loading rate (L_unloadr) is PG calculated by the second peak pressure and the end pressure of the stance phase. The L_valleyPG represents the sum of the absolute values of the gradient at each sampling point between the 2 peaks for the total pressure curve, describing the degree of pressure variation of the 2 peaks. This section extracts a total of 50 features from both feet.



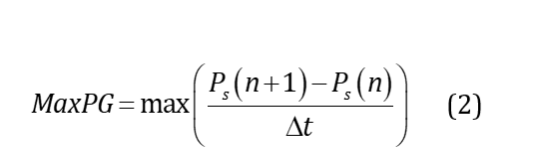





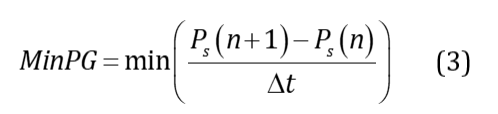



#### Temporal Features

As depicted in Figure 2, the gait cycle can be divided into the stance and swing phases. Taking the left foot as an example, the single-foot temporal features include the ratio of time between the stance and swing phases (L_t_st/sw), the gait cycle time (L_t_T), the ratio of stance phase to gait cycle time (L_t_st/T), and the proportion of the time to reach the first (L_t_t_1_/T) and second peaks (L_t_t_2_/T) in the total gait cycle time. Bipedal temporal features include single-limb support time and double-limb support time [35]. This section extracts a total of 12 temporal features.

#### Pressure Time Integral

By quantifying the accumulated pressure over the duration of the stance phase beneath discrete foot regions, the pressure time integral (PTI) depicts the total mechanical dose imparted to soft tissues during one step. However, PTI shows a high concordance with PPP [36]. To avoid redundancy, only the PTI of the global region is extracted here. PTI from heel strike to the first peak pressure (LPTI_1) and PTI of the stance phase (LPTI_st) were extracted from equation 4. In this part, a total of 4 left and right foot features were extracted.



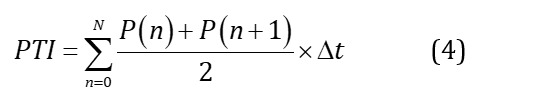



#### COP Features

COP is a commonly used dynamic parameter to track weight transfer. During the gait cycle from heel strike to toe-off, a series of coordinates for the COP trajectory can be obtained by calculating the weighted average of all pressure inputs acting on the foot, as defined by equation 5. Here x_s_ and y_s_ represent the sensor coordinates, which are converted into a unified coordinate system before computation, considering different shoe sizes [31]. Take the left foot during one step, for example, the mean and SD of COP trajectory in the medial-lateral direction (L_xcop_mean, L_xcop_std) and for anterior-posterior direction (L_ycop_mean, L_ycop_std) were calculated. Length of the COP trajectory (L_cop_len) can be calculated by equation 6. The resultant distance (RD) is the Euclidean distance between 2 points in COP coordinates. The mean (L_cop_MRD) and SD of RD (L_cop_SRD) can be calculated by equations 7 and 8, respectively [31]. In this part, 14 features of the left and right feet were extracted.



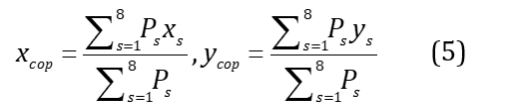





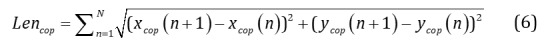





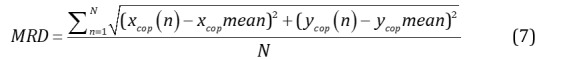





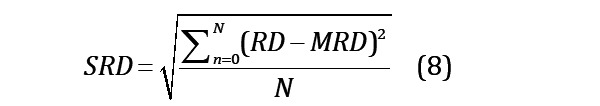



So far, a total of 54 single-foot features and 2 bipedal temporal features during one step have been extracted, and these will be averaged over 50 gait cycles. The following feature construction is to further expand the dimension of gait features through mathematical methods.

#### Symmetry Index

Asymmetrical gait patterns may be present in patients with KOA with functional impairment [37]. The symmetry index (SI) for the mentioned 54 single-foot features can be calculated by equation 9, where L_f_ and R_f_ represent the corresponding left and right foot features, respectively. In this part, 54 corresponding SI features are extracted and named with SI prefixes.



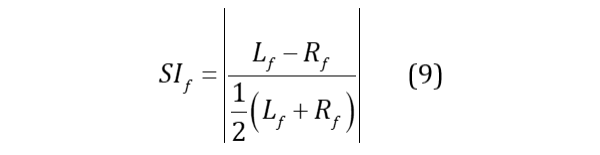



#### SD Feature

Patients with KOA often exhibit an unstable and highly variable gait pattern. Therefore, it is essential to extract the variability of relevant features across consecutive gait cycles. In this session, the SDs of 108 single foot features on both sides during 50 gait cycles were calculated and named with the suffix STD.

#### Weak Foot Feature

In previous studies [38], values reflecting lower performance were chosen from the features calculated separately for each leg. To further enhance the value of the extracted data variables and reduce the dependence of predictive models on extraction sides, this study refers to previous research on fall risk prediction in older adults [31] and extracts the weak foot features, that is, the features of the foot with weaker functional performance between the 2 feet. Features derived from the weaker side are posited to perhaps carry more predictive value for functional estimations. Weak foot features can be identified by anterior-posterior direction variability:



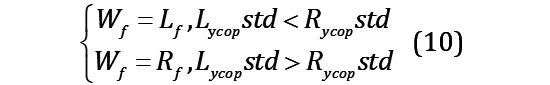



In this part, 108 weak-foot features named with the W prefix are extracted from the mean and SD of both foot single-foot features.

#### Physical Characteristics

Physical characteristics correlate with functional performance, so age, height, weight, and BMI are also included in the feature database.

All extracted features contained in the feature database are listed in Table 3. The related features of the left foot are named with the prefix L and the right foot with the prefix R.

**Table 3:** List of features.

Kinds of features		Features	Number
**Single foot features**			
	PPP^a^	L_s_PPP^b,c^, s ∈ (1,8)^d^; L_1-4_PPP^e^; L_5-6_PPP^f^; L_7-8_PPP^g^; L_peak1^h^; L_peak2^i^; L_4/2_PPP^j^; L_8/7_PPP^k^; R_s_PPP^l^, s ∈ (1,8); R_1-4_PPP; R_5-6_PPP; R_7-8_PPP; R_peak1; R_peak2; R_4/2_PPP; R_8/7_PPP	15×2
	PG^m^	L_s_MaxPG^m^, L_s_MinPG^o^, s ∈ (1,8); L_1-4_MaxPG^p^; L_1-4_MinPG^q^; L_5-6_MaxPG^r^; L_5-6_MinPG^s^; L_7-8_MaxPG^t^; L_7-8_MinPG^u^; L_loadr^v^; L_unloadr^w^; L_valleyPG^x^; R_s_MaxPG, R_s_MinPG, s ∈ (1,8); R_1-4_MaxPG; R_1-4_MinPG; R_5-6_MaxPG; R_5-6_MinPG; R_7-8_MaxPG; R_7-8_MinPG; R_loadr; R_unloadr; R_valleyPG	25×2
	Temporal	L_t_st/sw^y-aa^; L_t_T^ab^; L_t_st/T; L_t_t_1_/T^ac^; L_t_t_2_/T^ad^; R_t_st/sw; R_t_T; R_t_st/T; R_t_t_1_/T; R_t_t_2_/T	5×2
	PTI^ae^	LPTI_1; LPTI_st; RPTI_1; RPTI_st	2×2
	COP^af^	L_xcop_mean^ag^; L_xcop_std^ah^; L_ycop_mean^ai^; L_ycop_std; L_cop_len^aj^; L_cop_MRD^ak^; L_cop_SRD^al^; R_xcop_mean; R_xcop_std; R_ycop_mean; R_ycop_std; R_cop_len; R_cop_MRD; R_cop_SRD	7×2
**Bipedal features**		Single limb support time; double limb support time	2
**Symmetry index features**		SI_f_^am^	54
**SD features**		L_f__STD; R_f__STD	108
**Weak foot features**		W_f_^an^; W_f__STD^ao^	108
**Physical characteristics**		Age; height; weight; BMI	4

^a^PPP: peak plantar pressure.

^b^L: left foot.

^c^_s_: sensor.

^d^s: sensor.

^e^_1-4:_ forefoot.

^f^_5-6_: middle foot.

^g^_7-8_: heel.

^h^peak1: first vertical force peak.

^i^peak2: second vertical force peak.

^j^_4/2_PPP represents the ratio of peak pressure in the lateral and medial regions of the forefoot.

^k^_8/7_PPP represents the ratio of peak pressure in the lateral and medial regions of the heel.

^l^R: right foot.

^m^PG: pressure gradient.

^n^Min: minimum.

^o^Max: maximum.

^p^_1-4_MaxPG represents the maximum gradient change upward when the center of gravity shifts to the forefoot.

^q^_1-4_MinPG signifies the maximum negative gradient change when the toes lift off the ground, corresponding to the downward pressure curve.

^r^_5-6_MaxPG in the midfoot region reflect the pressure change during the foot flat phase.

^s^_5-6_MinPG in the midfoot region reflect the pressure change during the heel-off phase.

^t^_7-8_MaxPG in the heel region denote the changes during the heel strike phase.

^u^_7-8_MinPG: in the heel region denote the changes during the foot flat phase.

^v^loadr: loading rate.

^w^unloadr: off-loading rate.

^x^valleyPG: the sum of the absolute values of the gradient at each sampling point between the 2 peaks for the total pressure curve, describing the degree of pressure variation of the 2 peaks.

^y^_t_: time.

^z^st: stance phase.

^aa^sw: swing phase.

^ab^T: gait cycle time.

^ac^t_1_: first peak.

^ad^t_1_: second peak.

^ae^PTI: pressure time integral.

^af^COP: center of pressure.

^ag^_x_: medial-lateral direction.

^ah^std: SD.

^ai^_y_: anterior-posterior direction.

^aj^len: length of the center of pressure trajectory.

^ak^MRD: mean of resultant distance.

^al^SRD: SD of resultant distance.

^am^SI_f_: The corresponding symmetry index features of both feet.

^an^W_f_: mean value of weak foot features.

^ao^W_f__STD: SD of weak foot features.

### Feature Selection

#### Overview

**Figure 3:**
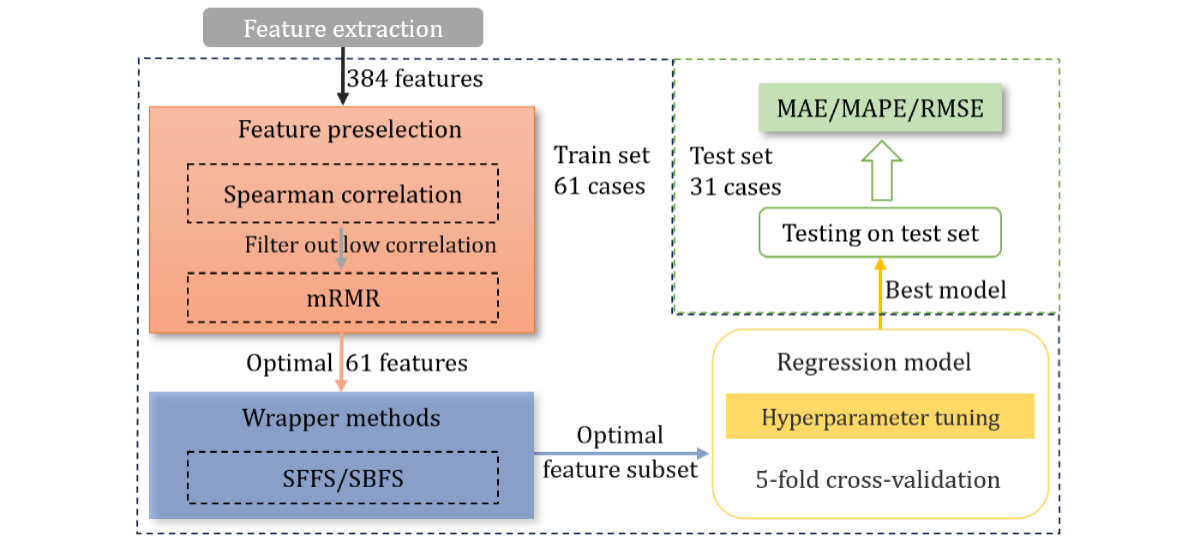
Pipeline of feature selection and model development. MAE: mean absolute error; MAPE: mean absolute percentage error; mRMR: Minimum Redundancy and Maximum Relevance; RMSE: root mean squared error; SBFS: Sequential Backward Floating Selection; SFFS: Sequential Forward Floating Selection.

In the feature extraction process, a total of 380 gait features and 4 physical characteristics were extracted to form the feature database. For machine learning model training, high-dimensional data not only increases computational workload but also results in severe overfitting, leading to poor generalization performance of the model. Therefore, before developing regression prediction models, it is necessary to undertake feature selection from the extensive feature database to alleviate the curse of dimensionality [39]. If the data of all participants is used during the feature selection process, it may lead to premature use of the testing set in feature selection, causing information leakage and inflating the model’s performance. Therefore, before feature selection, we used a holdout method by randomly partitioning 33% (31 cases) of the data as an external testing set, while the remaining 67% (61 cases) of the data is designated as the training set for feature selection, model development, and performance comparison. The testing set is strictly excluded from both the feature selection and model development processes, ensuring that the model exhibits objective and genuine generalization performance when confronted with new participants. Figure 3 illustrates the pipeline of feature selection and model development.

There are various methods for feature selection, mainly categorized into filter, embedded, and wrapper methods. This study used correlation analysis and the filter method, specifically the Minimum Redundancy and Maximum Relevance (mRMR) [40], as a preliminary feature selection. Subsequently, the wrapper methods were used to further determine the optimal feature subset. The stepwise reduction of the feature space through the combination of multiple methods proved effective in identifying valuable features. Meanwhile, principal component analysis [41], a popular feature dimensionality reduction method, was used as a benchmark for comparison to evaluate the effectiveness of the stepwise feature selection approach. The same machine learning model was trained on the reduced datasets and cross-validation was used to evaluate the performance of different methods.

#### Spearman Correlation Coefficient

Conducting Spearman correlation analysis between each feature and the corresponding task labels on the training set, features with low correlation (|r|<0.2) are eliminated to enhance computational efficiency.

#### About mRMR

Owing to the possible information redundancy between single-foot features and weak-foot features, it is not conducive to the speed, accuracy, and interpretability of the training results. The mRMR is a minimal-optimal feature selection algorithm that can find a subset of features in a machine learning task that has the greatest correlation with the target variables and the least redundancy between them [40]. Choose the optimal feature for the next feature selection, ensuring that its number does not exceed the number of samples in the training set.

#### Wrapper Methods

After feature preselection using statistical analysis methods, the wrapper methods based on the machine learning model are used for a more comprehensive feature selection. The wrapper methods determine the optimal feature subset through the average performance of cross-validation. This paper uses Sequential Feature Selection algorithms, including Sequential Forward Floating Selection and Sequential Backward Floating Selection, to automatically determine the optimal feature subset based on their impact on the performance of a user-defined model [42]. These 2 algorithms are implemented using *mlxtend* (version 0.20.0 for Python 3.7; Python Software Foundation) [43].

### Regression Model Development and Evaluation

After stepwise feature selection, a subset of features most relevant to the current problem is identified for developing machine learning models. The models considered include linear regression (LR), support vector machine (SVM), random forest (RF), Adaptive Boosting (AdaBoost), Extreme Gradient Boosting (XGBoost), and Light Gradient Boosting Machine (LGBM). Hyperparametric tuning of each model was performed using Optuna (Preferred Networks, Inc) [44] and 5-fold cross-validation was used to evaluate the results.

**Figure 4:**
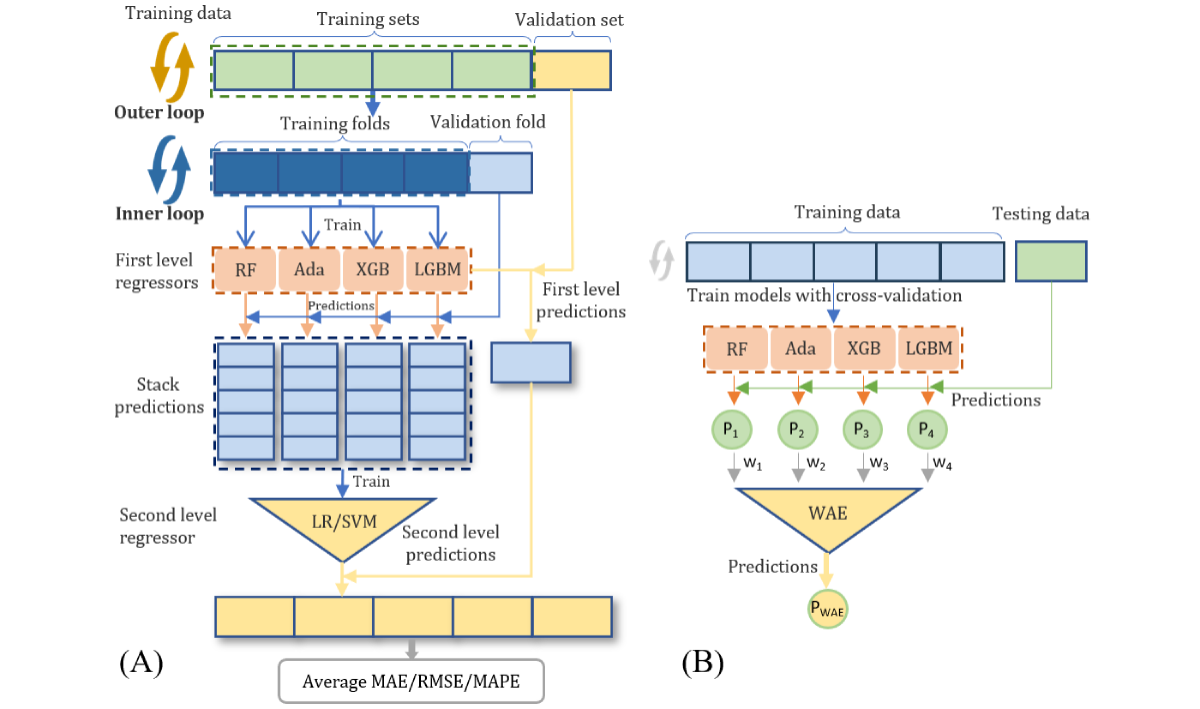
(A) Flowchart of stacking regressors with nested 5-fold cross-validation. (B) The prediction process of the WAE model. Ada: Adaptive Boosting; LGBM: Light Gradient Boosting Machine; LR: linear regression; MAE: mean absolute error; P: prediction; RF: random forest; RMSE: root mean squared error; MAPE: mean absolute percentage error; SVM: support vector machine; W: weight; WAE: weighted average ensemble; XGB: Extreme Gradient Boosting.

Based on the training results of each model and referring to previous studies [45], the 2-level ensemble learning model was constructed using the stacking regression method [46], as shown in Figure 4A. The training data were randomly partitioned into 5 mutually exclusive subsets, 4 of which were used as 5-fold cross-validation of the inner loop to train the models. At the first level, 4 decision-tree-based regressors including RF, AdaBoost, XGBoost, and LGBM were used to fit the training folds, respectively, and then predict the validation fold. The predictions from these 5 rounds were stacked to form the input features for the second-level regressor, which uses a simple model such as LR or SVM. The validation set of the external loop is used to evaluate the performance of the stacking model and hyperparameter tuning. Additionally, for comparison purposes, simple average ensemble (SAE) and weighted average ensemble (WAE) were also adopted to construct 2-level models for these 4 tree-based models. The final prediction of the SAE model is obtained by taking the average of the predictions from all individual models, while WAE assigns different weights to the predictions of each model according to their performance, allowing models with better performance to have a greater influence on the final prediction, as shown in Figure 4B. The ensemble model can reduce variance, enhance robustness, and improve generalizability by combining the prediction results from multiple models.

Mean absolute error (MAE), mean absolute percentage error (MAPE), and root mean squared error (RMSE) were used as regression performance metrics to evaluate the results of different models [47,48]. To avoid deceiving performance caused by data bias, the average of the known training set labels is used as the prediction of the unknown test set to calculate these metrics as the performance of the baseline model. The improvement of each metric for each model relative to the baseline model is calculated and normalized into a regress relative index (RI) to comprehensively evaluate the model performance [49,50], as shown in equation 11.



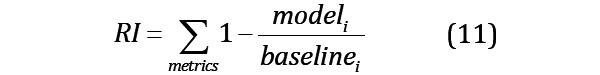



Where *model_i_* and *baseline_i_* represent the performance metrics corresponding to each model and the baseline model, respectively.

Models with high RI values will be considered as candidate regressors for 2-level ensemble learning models. The contribution of each model in WAE prediction was weighted according to the proportion of their RI value. The final predicted value ŷ_WAE_ of the WAE model can be calculated by equation 12.



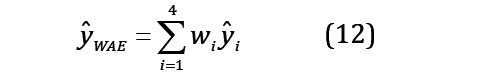



### Ethical Considerations

All experimental procedures were approved by the institutional review board of Zhujiang Hospital of Southern Medical University (IRB 2019-KY-016-02). This study ensures informed consent with the right to withdraw. Participants’ privacy is safeguarded through data anonymization. Compensation for human subjects involved a payment of CN ¥200 (CN ¥1=US $0.14) per individual.

## Results

### Feature Selection Results

Table 4 shows the benchmark experimental results of feature selection, comparing the performance of various algorithms using the RF regressor as the base model, and it can be seen that the method using stepwise feature selection performs better than the principal component analysis algorithm in both tasks.

**Figure 5:**
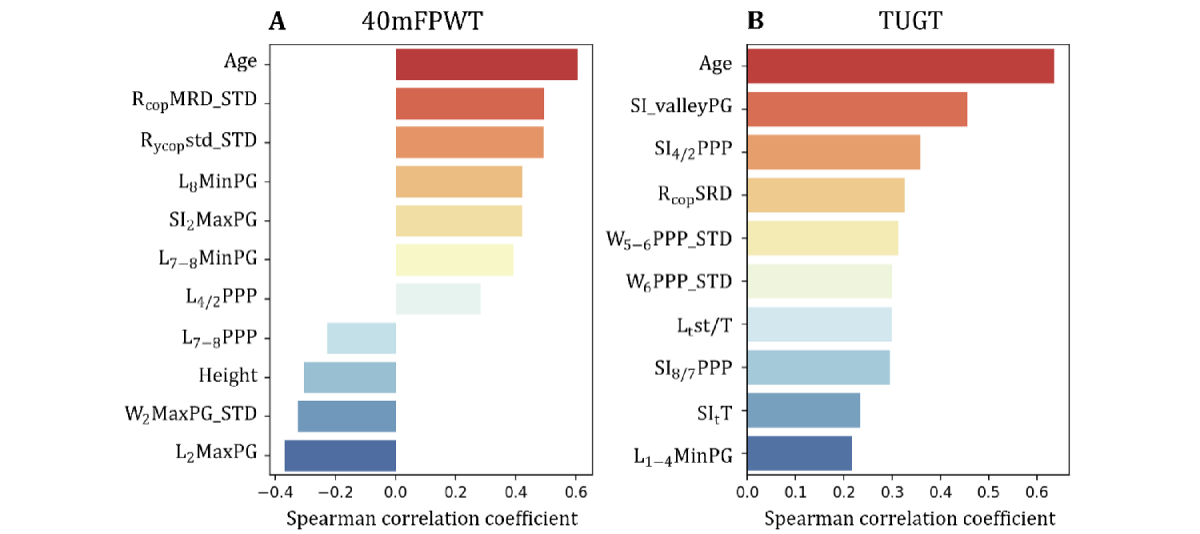
The optimized feature subsets and Spearman correlation coefficients for (A) the 40mFPWT task and (B) the TUGT task. _1-4_MinPG: the maximum negative gradient change when the toes lift off the ground, corresponding to the downward pressure curve; _2_: medial region of the forefoot; _4/2_PPP: ratio of peak pressure in the lateral and medial regions of the forefoot; 40mFPWT: 40-m fast-paced walk test; _5-6_: middle foot; _7-8_: heel; _7-8_MinPG: changes during the foot flat phase in the heel region; _8_: lateral region of the heel; _8/7_PPP: ratio of peak pressure in the lateral and medial regions of the heel; COP: center of pressure; L: left foot; Max: maximum; Min: minimum; MRD: mean of resultant distance; PG: pressure gradient; PPP: peak plantar pressure; R: right foot; SI: symmetry index; SRD: SD of resultant distance; st: stance phase; std: SD; STD: SD; _t_: time; T: gait cycle time; TUGT: timed up-and-go test; valley: the sum of the absolute values of the gradient at each sampling point between the 2 peaks for the total pressure curve, describing the degree of pressure variation of the 2 peaks; W: weight; _y_: anterior-posterior direction.

After applying the Spearman correlation coefficient to filter out low-correlation noise features, the 40mFPWT task retained 161 features and the TUGT task retained 131 features. Then select an optimal subset of 61 features with mRMR, which was set to not exceed the number of training samples. For 40mFPWT, the subset of 11 features identified by the Sequential Backward Floating Selection method yields the best performance. For TUGT, the optimal feature subset determined by the Sequential Forward Floating Selection method consists of 10 features. The optimized feature subsets and Spearman correlation coefficients for both tasks are depicted in Figure 5.

**Table 4:** Results of benchmark experiment on the feature selection algorithm.

Tasks and methods			MAE^a^ (s)		MAPE^b^ (%)		RMSE^c^ (s)
**40** **mFPWT^d^**							
	PCA^e^	4.638		15.223		6.023	
	SFFS^f^	2.813		9.167		3.791	
	SBFS^g^	2.698		8.854		3.66	
**TUGT^h^**							
	PCA	2.063		17.121		2.771	
	SFFS	1.589		12.801		2.285	
	SBFS	1.643		13.38		2.236	

^a^MAE: mean absolute error.

^b^MAPE: mean absolute percentage error.

^c^RMSE: root mean squared error.

^d^40mFPWT: 40-m fast-paced walk test.

^e^PCA: principal component analysis.

^f^SFFS: Sequential Forward Floating Selection.

^g^SBFS: Sequential Backward Floating Selection.

^h^TUGT: timed up-and-go test.

### Evaluation of Machine Learning Regression Model

After feature selection, six regression models were trained and their hyperparameters were tuned using Optuna with 5-fold cross-validation for both the 40mFPWT and TUGT tasks, respectively. RF, AdaBoost, XGBoost, and LGBM were used as first-level regressors for the stacked model, and the performance of LR and SVM as second-level regressors were compared, respectively. SAE combines the predictions from 4 trained tree-based models equally, while WAE weighs the contribution of each ensemble member proportionally based on RI value. The performance of each model compared with the baseline model is shown in Table 5 for 40mFPWT and Table 6 for TUGT. Among individual models, the LGBM model achieved the best predictive performance in the 40mFPWT task, while the XGBoost model performed best in the TUGT task. The performance of the stacking model using SVM as the second-level regressor is better than that using LR in both tasks. The average integration model using the WAE strategy has better performance than that using SAE in both tasks.

**Table 5:** Results of cross-validation of regression models in the 40mFPWT^a^ training set.

Model		MAE^b^ (s)	MAPE^c^ (%)	RMSE^d^ (s)	RI^e^
**Baseline**		4.925	16.461	6.219	0
**Individual models**					
	LR^f^	3.793	12.426	4.966	0.676
	SVM^g^	3.318	10.653	4.526	0.951
	RF^h^	2.59	8.407	3.546	1.393
	AdaBoost^i^	2.541	8.137	3.546	1.42
	XGBoost^j^	2.495	7.758	3.527	1.455
	LGBM^k,l^	2.373^l^	7.473^l^	3.381^l^	1.521^l^
**Stacked model**					
	Stack (linear)	2.518	7.921	3.432	1.456
	Stack (SVM)^l^	2.223^l^	6.929^l^	3.152^l^	1.621^l^
**Average ensemble**					
	SAE^m^	2.345	7.42	3.334	1.537
	WAE^n,l^	2.34^l^	7.399^l^	3.329^l^	1.54^l^

^a^40mFPWT: 40-m fast-paced walk test.

^b^MAE: mean absolute error.

^c^MAPE: mean absolute percentage error.

^d^RMSE: root mean squared error.

^e^RI: relative index.

^f^LR: linear regression.

^g^SVM: support vector machine.

^h^RF: random forest.

^i^AdaBoost: Adaptive Boosting.

^j^XGBoost: Extreme Gradient Boosting.

^k^LGBM: Light Gradient Boosting Machine.

^l^the optimal result in the various model methods.

^m^SAE: simple average ensemble.

^n^WAE: weighted average ensemble.

**Table 6:** Results of cross-validation of regression models in the TUGT^a^ training set.

Model		MAE^b^ (s)	MAPE^c^ (%)	RMSE^d^ (s)	RI^e^
**Baseline**		2.475	20.969	3.267	0
**Individual model**					
	LR^f^	1.739	14.174	2.285	0.922
	SVM^g^	1.717	13.859	2.318	0.936
	RF^h^	1.538	12.396	2.16	1.126
	AdaBoost^i^	1.399	11.218	2.043	1.274
	XGBoost^j,k^	1.306^k^	10.365^k^	1.891^k^	1.399^k^
	LGBM^l^	1.397	11.012	1.944	1.315
**Stacked model**					
	Stack (linear)	1.473	11.775	2.033	1.221
	Stack (SVM)^k^	1.3^k^	10.2^k^	1.873^k^	1.415^k^
**Average ensemble**					
	SAE^m^	1.347	10.701	1.945	1.35
	WAE^n,k^	1.339^k^	10.633^k^	1.936^k^	1.359^k^

^a^TUGT: timed up-and-go test.

^b^MAE: mean absolute error.

^c^MAPE: mean absolute percentage error.

^d^RMSE: root mean squared error.

^e^RI: relative index.

^f^LR: linear regression.

^g^SVM: support vector machine.

^h^RF: random forest.

^i^AdaBoost: Adaptive Boosting.

^j^XGBoost: Extreme Gradient Boosting.

^k^the optimal result in the various model methods.

^l^LGBM: Light Gradient Boosting Machine.

^m^SAE: simple average ensemble.

^n^WAE: weighted average ensemble.

### Prediction Outcomes for Functional Tests

The best-performing trained models obtained from optimizing hyperparameters on the training set were used to generate predictions for the holdout test set, including the individual model, stacked model, and average ensemble model. The results in the holdout test set for the 40mFPWT are shown in Table 7 and for the TUGT are shown in Table 8. Among all models, the WAE model using 4 tree-based models has the best generalization performance in the test set, with MAE of 2.686 seconds, MAPE of 9.602%, and RMSE of 3.316 seconds for the prediction of 40mFPWT, and for TUGT with MAE of 1.280 seconds, MAPE of 12.389%, and RMSE of 1.905 seconds.

**Table 7:** Results of the models in the 40mFPWT^a^ testing set.

Model	MAE^b^ (s)	MAPE^c^ (%)	RMSE^d^ (s)
Baseline	3.9	14.664	4.515
LGBM^e^	2.918	10.404	3.578
Stack (SVM^f^)	2.787	9.77	3.514
WAE^g^	2.686	9.602	3.316

^a^40mFPWT: 40-m fast-paced walk test.

^b^MAE: mean absolute error.

^c^MAPE: mean absolute percentage error.

^d^RMSE: root mean squared error.

^e^LGBM: Light Gradient Boosting Machine.

^f^SVM: support vector machine.

^g^WAE: weighted average ensemble.

**Table 8:** Results of the models in the TUGT^a^ testing set.

Model	MAE^b^ (s)	MAPE^c^ (%)	RMSE^d^ (s)
Baseline	1.608	15.637	1.999
XGBoost^e^	1.437	13.714	2.128
Stack (SVM^f^)	1.465	13.91	2.1
WAE^g^	1.280	12.389	1.905

^a^TUGT: timed up-and-go test.

^b^MAE: mean absolute error.

^c^MAPE: mean absolute percentage error.

^d^RMSE: root mean squared error.

^e^XGBoost: Extreme Gradient Boosting.

^f^SVM: support vector machine.

^g^WAE: weighted average ensemble.

## Discussion

### Principal Findings

This study developed a functional evaluation model using multidimensional plantar pressure features to predict the functional performance of patients with KOA. The plantar pressure data collected by the shoe system were preprocessed through feature engineering. These features were then input into the trained model to enable prediction and thereby realize functional assessment and monitoring of patients with KOA.

The results of feature selection indicated that age was the most relevant predictor of functional performance on the 2 tasks. It is reasonable that higher age correlated with a longer duration to finish the function tests, and therefore poorer function (Figure 5). Notably, 4 features showed negative correlations with 40mFPWT outcomes (Figure 5A). Specifically, higher values of the L_7-8_PPP feature were associated with better functionality, aligned with previous findings that individuals with KOA tend to exhibit diminished plantar pressure in the heel region [25,26]. Four SI features were selected for the TUGT task. This is likely because diseases or impairments that impact proprioception or postural stability could thereby influence balance performance on the TUGT by altering one’s symmetry [51]. The results showed that plantar pressure–derived features in the forefoot and rearfoot regions, specifically PG and PPP values, exhibited relatively strong correlations with functional test outcomes. In addition, COP-derived features, SI features, and weak foot features were selected and exhibited relatively strong correlations with functional test outcomes. These observed relationships are biologically plausible and concordant with existing understandings of pathological gait patterns in populations with KOA.

The performance of the baseline model, which uses the mean of known labels in the training set as predictions for the test set, provides an unbiased evaluation metric without issues of overfitting or overoptimism. The model-free nature of this design ensures a fair assessment of predictive gains attributable to model architecture rather than data traits. The RI value of each model was obtained by calculating the sum of the improvement of each performance metric relative to the baseline. A higher RI value represents better overall model performance. The results demonstrate that tree-based models outperform LR and SVM models significantly (Tables 3 and 4). Using SVM as the second-level regressor in the stacked model yields better results than LR, primarily attributable to SVM’s ability to handle nonlinear relationships and demonstrate robustness against outliers in the data. Within the averaged ensemble framework, the WAE model outperformed the SAE model, possibly due to its weighted aggregation mechanism. The WAE model produced superior outcomes by assigning higher weights to individual models with higher predictive power, thereby generating more refined ensemble predictions.

Due to feature selection being performed on the training set, the models may still overfit the training set even with the use of cross-validation techniques, resulting in overly optimistic performance estimates. Therefore, in general, the model performs better on a training set than on a holdout test set containing unknown samples. The performance on the test set provides a more robust evaluation of the models’ generalization capability when encountering new data. The results indicate that the WAE model demonstrated the best generalization performance in both tasks, rather than the stacked model that used SVM as the second-level regressor (Tables 5 and 6). This aligns with the principle of Occam razor in model selection which is to prefer the more parsimonious model when performance is otherwise comparable [52].

Based on the prediction results from the 2 functional tests, the features we extracted appear to have a reasonably close correlation with functional performance. The models demonstrated good generalization for predicting traditional clinical function tests. In the future, the model could be integrated into a terminal application to longitudinally monitor patients’ functional status. The identified plantar pressure features could serve as an evaluation tool to guide the rehabilitation and assessment of progress for patients with KOA, offering clear advantages per time efficiency, longitudinal documentation, and accuracy compared to conventional functional tests.

The findings of this study have significant clinical implications for the management and rehabilitation of patients with KOA. The proposed techniques enable continuous, real-time monitoring of patients’ functional status beyond clinical settings. This capability facilitates more personalized and timely interventions. By accurately assessing functional performance, patients gain greater insight into their condition thereby improving overall management. Additionally, the system’s ability to decrease the frequency of hospital visits and extensive clinical assessments contributes to cost-effectiveness, alleviating the burden on health care systems.

### Limitations and Strengths

Several limitations of this study should be noted. First, this study’s cohort consisted exclusively of patients diagnosed with KOA without the inclusion of data from healthy control participants for comparative analysis. The validity of the model prediction is limited to the patients’ population with KOA. The participant data of this study are mostly female, and the validity of the model may be biased toward female patients. Second, this was a cross-sectional study without long-term longitudinal monitoring of patients in their daily living environments. Third, it only conducted a feasibility study on functional estimation based on wearable plantar pressure features for 2 clinical functional tests recommended by OARSI for KOA. Hence, the proposed techniques require further validation in larger prospective cohorts and preferably multicenter trials to corroborate generalizability.

Despite its limitations, we believe that this wearable plantar pressure technique captures objective quantitative indicators of functional status and has great application value. The preliminary findings indicate this methodology holds promise for enabling remote, quantitative monitoring of rehabilitation progress over time. Further work will refine the system for broader clinical application and validation.

### Conclusions

This study aims to develop a lower extremity motor function evaluation model for patients with KOA based on multidimensional gait features, which was suitable for a wearable plantar pressure measurement system. The average performance and variability of left and right foot features were extracted from the raw plantar pressure data. An extensive feature database including 380 gait features and 4 physical characteristics was established by mathematical methodologies. Optimal feature subsets for both tasks are selected after stepwise feature selection including Spearman correlation coefficient, mRMR, and wrapper methods. Individual regression models and a 2-level ensemble learning model were trained for the 40mFPWT and TUGT tasks, respectively. The WAE model that weighs the contribution of each ensemble member proportionally based on RI value has the best performance in the testing set, with an MAE of 2.686 seconds, MAPE of 9.602%, and RMSE of 3.316 seconds for the 40mFPWT and an MAE of 1.280 seconds, MAPE of 12.389%, and RMSE of 1.905 seconds for the TUGT. The proposed technique has the potential to be a novel approach for objectively quantifying the functionally dependent gait features, which could be developed as a tool for the rehabilitation evaluation of motor function in individuals with KOA. This study fills the vacancy in dynamic functional assessment for patients with KOA based on wearable devices. In future work, a variety of sensing technologies will be integrated to evaluate and predict more functional tests, providing more accurate and scientific support in fields such as sports medicine and rehabilitation therapy.

## Abbreviations

40mFPWT: 40-m fast-paced walk test
AdaBoost: Adaptive Boosting
COP: center of pressure
KOA: knee osteoarthritis
LGBM: Light Gradient Boosting Machine
LR: linear regression
MAE: mean absolute error
MAPE: mean absolute percentage error
mRMR: Minimum Redundancy and Maximum Relevance
OARSI: Osteoarthritis Research Society International
PBM: performance-based measure
PG: pressure gradient
PPP: peak plantar pressure
PRO: patient-reported outcome
PTI: pressure time integral
RD: resultant distance
RF: random forest
RI: relative index
RMSE: root mean squared error
SAE: simple average ensemble
SI: symmetry index
SVM: support vector machine
TUGT: timed up-and-go test
WAE: weighted average ensemble
WOMAC: Western Ontario and McMaster Universities Osteoarthritis Index
XGBoost: Extreme Gradient Boosting
